# Trends in AIDS Deaths, New Infections and ART Coverage in the Top 30 Countries with the Highest AIDS Mortality Burden; 1990–2013

**DOI:** 10.1371/journal.pone.0131353

**Published:** 2015-07-06

**Authors:** Reuben Granich, Somya Gupta, Bradley Hersh, Brian Williams, Julio Montaner, Benjamin Young, José M. Zuniga

**Affiliations:** 1 International Association of Providers of AIDS Care, Washington D.C., United States of America; 2 Joint United Nations Programme on HIV/AIDS (UNAIDS), Geneva, Switzerland; 3 Wits Reproductive Health Institute, University of the Witwatersrand, Johannesburg, South Africa; 4 British Columbia Centre for HIV/AIDS Excellence, Vancouver, British Columbia, Canada; University of Athens, Medical School, GREECE

## Abstract

**Background:**

Antiretroviral therapy (ART) prevents human immunodeficiency virus (HIV) disease progression, mortality and transmission. We assess the impact of expanded HIV treatment for the prevention of Acquired Immunodeficiency Syndrome (AIDS)-related deaths and simulate four treatment scenarios for Nigeria and South Africa.

**Methods:**

For 1990–2013, we used the Joint United Nations Programme on HIV/AIDS (UNAIDS) database to examine trends in AIDS deaths, HIV incidence and prevalence, ART coverage, annual AIDS death rate, AIDS death-to-treatment and HIV infections to treatment ratios for the top 30 countries with the highest AIDS mortality burden and compare them with data from high-income countries. We projected the 1990–2020 AIDS deaths for Nigeria and South Africa using four treatment scenarios: 1) no ART; 2) maintaining current ART coverage; 3) 90% ART coverage based on 2013 World Health Organization (WHO) ART guidelines by 2020; and 4) reaching the United Nations 90-90-90 Target by 2020.

**Findings:**

In 2013, there were 1.3 million (1.1 million–1.6 million) AIDS deaths in the top 30 countries representing 87% of global AIDS deaths. Eight countries accounted for 58% of the global AIDS deaths; Nigeria and South Africa accounted for 27% of global AIDS deaths. The highest death rates per 1000 people living with HIV were in Central African Republic (91), South Sudan (82), Côte d’Ivoire (75), Cameroon (72) and Chad (71), nearly 8–10 times higher than the high-income countries. ART access in 2013 has averted as estimated 1,051,354 and 422,448 deaths in South Africa and Nigeria, respectively. Increasing ART coverage in these two countries to meet the proposed UN 90-90-90 Target by 2020 could avert 2.2 and 1.2 million deaths, respectively.

**Interpretation:**

Over the past decade the expansion of access to ART averted millions of deaths. Reaching the proposed UN 90-90-90 Target by 2020 will prevent additional morbidity, mortality and HIV transmission. Despite progress, high-burden countries will need to accelerate access to ART treatment to avert millions of premature AIDS deaths and new HIV infections.

## Introduction

In the 33 years since acquired immunodeficiency syndrome (AIDS) was first described in 1981 and the discovery of the Human Immunodeficiency Virus (HIV), considerable progress has been made in combating the epidemic.[1] There have been significant declines in the number of new HIV infections and AIDS-related deaths, however, HIV remains a major global public health problem.[2] An estimated 35 million (33.2 million–37.2 million) people are living with HIV, 2.1 million people (1.9 million–2.4 million) were newly infected with HIV and there were around 1.5 million (1.4 million–1.7 million) AIDS-related deaths in 2013.[3] Antiretroviral therapy (ART) prevents disease progression, opportunistic infections, tuberculosis (TB), mortality and HIV transmission and studies suggest that expanded access to treatment in some settings could lead to the eventual elimination of HIV.[4–11] From 1995 to 2013, ART averted an estimated 7.6 million AIDS-related deaths globally, including 4.8 million deaths in sub-Saharan Africa.[3]

Earlier diagnosis and treatment is essential to prevent immune degradation and HIV transmission.[4,5,11,12] In 2013, the World Health Organization (WHO) issued new HIV treatment guidelines that recommend ART initiation at CD4 count ≤500 cells/mm^3^ for adults and adolescents and irrespective of CD4 count for pregnant women, HIV-positive partners in serodiscordant couples, children younger than five and people with HIV and tuberculosis (TB) or hepatitis B coinfection.[13] Global implementation of these guidelines could avert millions of infections and deaths. [13] Although access to treatment reached 12.9 million people by the end of 2013, only 37% (35–39%) of the 35 million (33.2 million–37.2 million) people living with HIV were receiving ART.[3] In 2013, the Joint United Nations Programme on HIV/AIDS (UNAIDS) launched the *Treatment 2015* initiative to support countries to reach 15 million people with HIV treatment by 2015 as a stepping-stone to accelerate progress towards universal access.[14] The 2015 target of 15 million now appears to be within reach and at the 20^th^ International AIDS Conference in Melbourne, the Joint United Nations Programme on HIV/AIDS (UNAIDS) proposed a new post-2015 90-90-90 Target.[15] The new target, now formally adopted by the United Nations (UN) on September 25^th^ 2014 during United Nations General Assembly Special Session, proposes that by 2020 at least 90% of all people living with HIV globally will know their status, 90% of those diagnosed will have sustained access to high quality ART, and 90% of those on ART will be virally suppressed.[16] Mathematical modelling shows that meeting the UN 90-90-90 Target by 2020 will enable the virtual elimination of the HIV/AIDS pandemic by 2030, defined as a ≥90% decrease in disease burden (including morbidity, mortality and incidence) using 2010 levels as a baseline.[16] However, this will require accelerated efforts to achieve the UN 90-90-90 Target through focused urban, rural, regional and national HIV responses. We assess the impact of expanded HIV treatment for the prevention of AIDS-related deaths. We analyzed the available UNAIDS data to describe AIDS-related deaths, ART coverage and new HIV infections in 30 high AIDS mortality burden countries and compared it with data from eight high-income countries. We also explored the potential impact of reaching international treatment expansion targets in South Africa and Nigeria through the examination of four treatment expansion scenarios.

## Methodology

### Data sources

UNAIDS maintains a database of country-reported data, *AIDSinfo*, which includes HIV prevalence, incidence and AIDS-related mortality estimates, most of them generated by Spectrum software.[17,18] The mortality estimate and uncertainty bounds calculation methodology is fully described elsewhere [17,18] and takes into consideration parameters such as ART coverage. We analysed the available country-level data for the top 30 countries representing 87% of burden of AIDS deaths in 2013 and compared them with data from eight high-income countries. We present analyses for South Africa and Nigeria–two countries with the largest HIV epidemics but with different trends of AIDS-related deaths over time.

### Indicators

ART coverage is defined as people reported to be on treatment at the end of the year divided by the estimated number of people living with HIV in the same year. We calculated two additional indicators—the annual AIDS death rate and the AIDS death-to-treatment ratio. The annual AIDS death rate is estimated as AIDS deaths per estimated 1000 people living with HIV. The “AIDS death to treatment” ratio was derived by dividing the estimated annual deaths by the number of people placed on ART during the same year. The number of people newly placed on ART is estimated as the difference in reported people on treatment between years. The “AIDS death to treatment tipping point” reflects program progress and is defined as when the number of estimated annual AIDS deaths is equal to the number of people put on treatment during the same time period. Similarly, the “new HIV infections to treatment tipping point” ratio was calculated by dividing the estimated new HIV infections by the number of people placed on ART during the same year.[19] Placing people on ART prevents deaths and HIV transmission, and as a proxy for progress towards targets, the program objective during expansion of access to treatment is to place more people on ART than those who die of AIDS-related causes or acquire HIV infection.

### Projections and scenarios for Nigeria and South Africa

We used the AIDS Impact Model (AIM) in Spectrum/EPP software (version 5.03) to project annual AIDS deaths during 1990–2020 for Nigeria and South Africa under four scenarios: (1) *No ART Scenario* which estimated AIDS deaths in the absence of treatment (including antiretroviral prophylaxis for prevention of mother-to-child transmission); (2) *Current ART Coverage Scenario* which projected the number of deaths assuming that current level of ART coverage (among people eligible for ART according to national guidelines) is maintained until 2020; (3) *2013 WHO Guidelines Scenario* which projected AIDS deaths under the assumption that countries implement the 2013 WHO guidelines at the end of 2013 and achieve 90% ART coverage using the estimated number of people eligible per 2013 WHO ART initiation criteria as a denominator by 2020; and 4) UN 90-90-90 Target *Scenario* i.e. reaching UN 90-90-90 Target or 73% of people living with HIV virally suppressed by 2020.

## Results

### Trends in AIDS deaths in the top 30 countries

**Fig 1:**
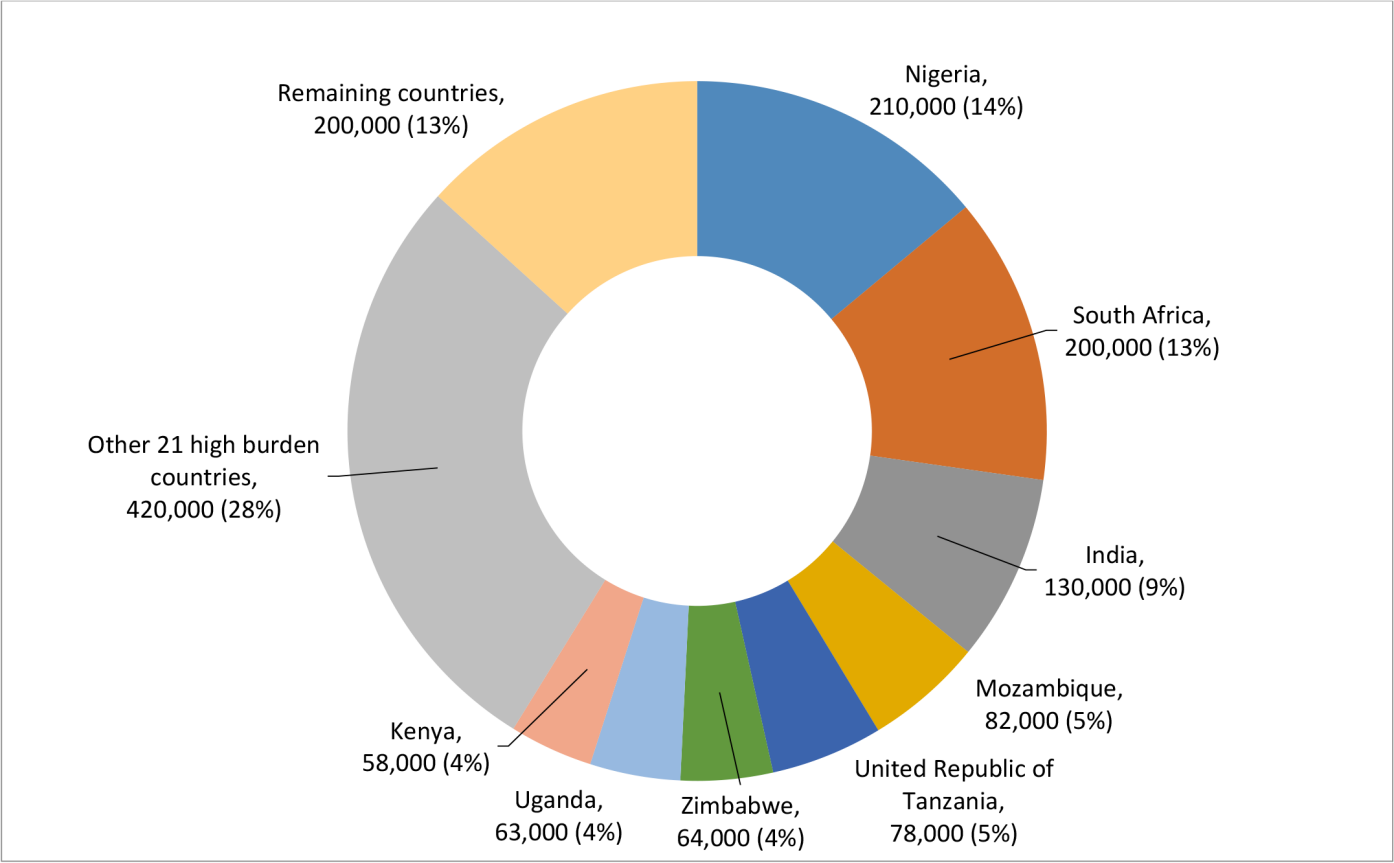
Estimated annual AIDS-related deaths by country, 2013. Number and percentage of estimated 1.5 million (1.4 million–1.7 million) global AIDS-related deaths in the eight highest burden countries for 2013.

Of an estimated 1.5 million (1.4 million–1.7 million) AIDS-related deaths globally in 2013, the top 30 countries with the highest AIDS mortality in 2013 accounted for 1.3 million (1.1 million–1.6 million) or 87% of total AIDS-related deaths. All but three countries (Brazil, Ukraine and Haiti) belong to two regions: Sub-Saharan Africa and Asia and the Pacific, the regions with the highest numbers of AIDS-related deaths. Only eight countries (Nigeria, South Africa, India, Mozambique, Tanzania, Zimbabwe, Uganda and Kenya) accounted for 58% of the global AIDS-related deaths in 2013 (**Fig 1**). The estimated annual AIDS-related deaths in the top 30 countries rose from 260,000 (200,000–360,000) in 1990 to peak at 2.1 million (1.8 million–2.3 million) in 2005 (**Data not shown**). Since 2005, AIDS deaths have been declining, representing a 36% decline in annual estimated AIDS deaths. Compared to the new HIV infections, which peaked at 3.1 million (2.7 million–3.6 million) in 1997, the peak in AIDS-related deaths occurred nearly a decade later.

Between 2005 and 2013, there has been a >50% decline in AIDS deaths in nine countries (Ethiopia, Ghana, Haiti, Kenya, Malawi, Namibia, Thailand, Zambia and Zimbabwe) representing 19% [280,000 (240,000–340,000)] of global AIDS-related deaths. Treatment in these countries increased from around 310,000 people on ART in 2005 to 3.2 million in 2013 [46% (43%-49%) of people living with HIV]. Significant decline in AIDS deaths was also seen in highest AIDS mortality burdened countries such as South Africa, India and Tanzania. Four countries (Indonesia, Malaysia, Mozambique and South Sudan) representing 9% [130,000 (96,000–180,000)] of global deaths have experienced a 42% increase in AIDS-related deaths between 2005 and 2013. In 2013, the number of reported people on ART in these countries was 568,968 [24% (19%-30%) of people living with HIV].

### AIDS deaths rate and AIDS death to treatment ratio

**Fig 2:**
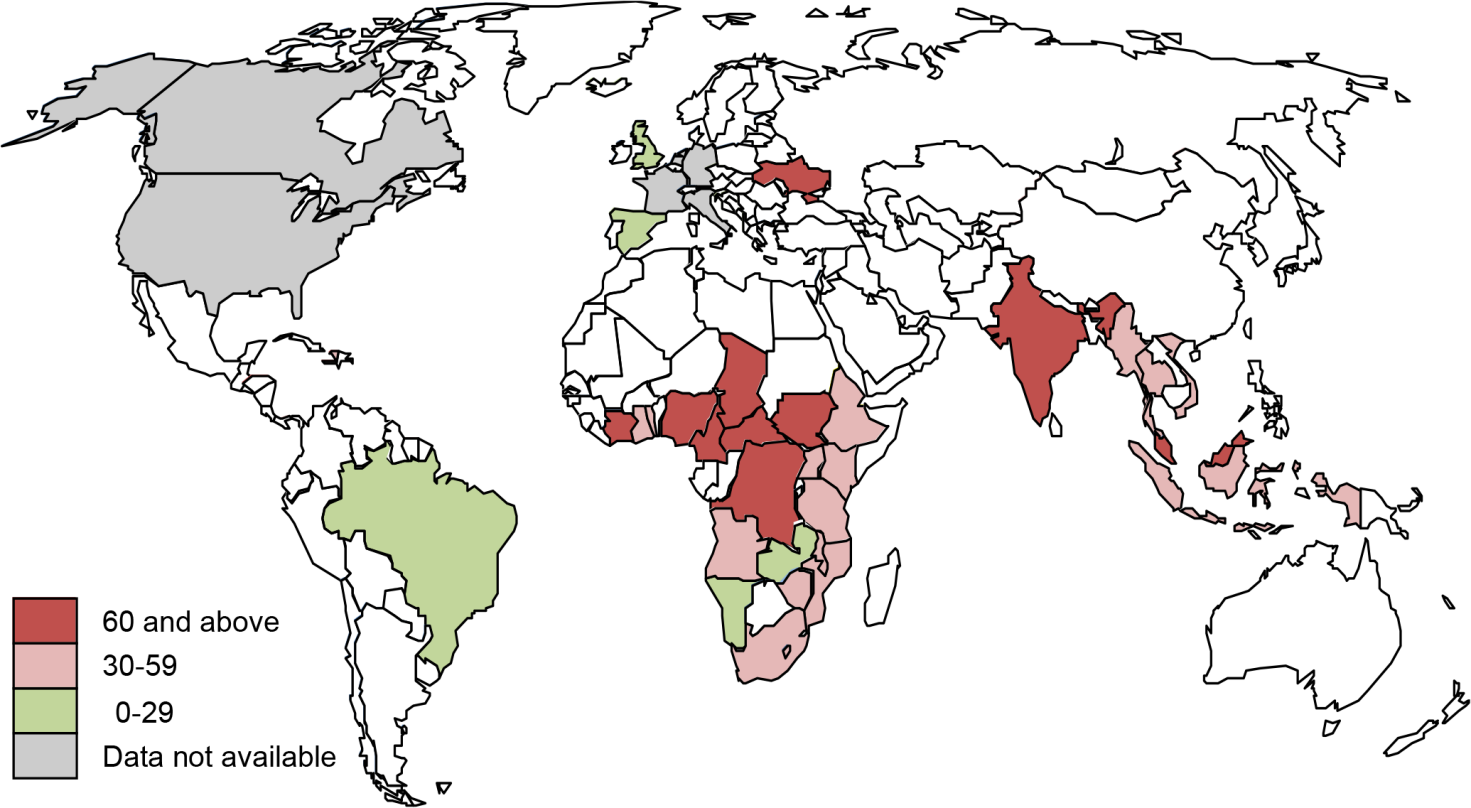
Global AIDS-related death rate per 1000 people living with HIV in 2013. Map of AIDS death rate per 1000 people living with HIV for 2013 for 30 high AIDS mortality burden countries and eight high-income countries. UNAIDS AIDS-related deaths estimates for 2013 are not available for countries shown in grey. Countries in white were not included in analyses.

**Fig 3:**
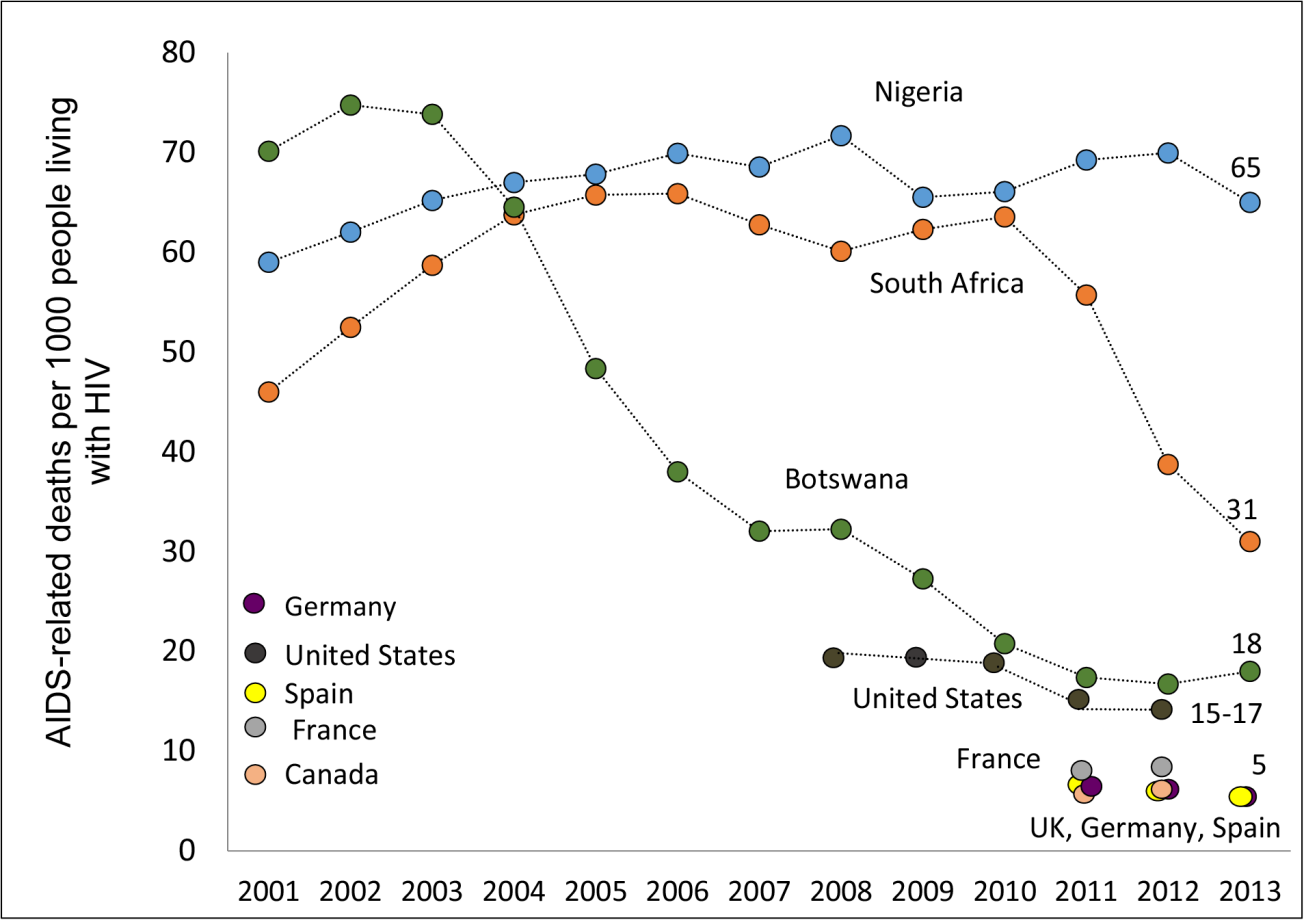
Trends in estimated death rate per 1000 people living with HIV, 2001–2013. Comparison of trends of estimated AIDS-related death rate per 1000 people living with HIV for selected high, middle and low-income countries from 2001 to 2013. Data from UNAIDS *AIDSinfo* data tool,^18^ the 2012 and 2013 UNAIDS Global Reports^2^ and Centers for Disease Control and Prevention Diagnoses of HIV Infection in the United States and Dependent Areas, 2011: HIV Surveillance Report, Volume 23.

Of the 30 countries with the highest AIDS-related death burden, Central African Republic (91 per 1000 people living with HIV), South Sudan (82 per 1000), Côte d’Ivoire (75 per 1000), Cameroon (72 per 1000) and Chad (71 per 1000) had the highest death rates (**Table 1, Fig 2**). The largest number of estimated AIDS-related deaths in 2013 was in Nigeria and South Africa; the death rate was 65 and 31 per 1000 people living with HIV, respectively. A comparison of death rates for selected high-, low- and middle-income countries demonstrates significant higher death rates in South Africa [31 per 1000] and Nigeria [65 per 1000] when compared with countries in North America and Europe (**Fig 3**). Additionally, between 2001 and 2013 the estimated annual death rate in Botswana decreased by 76% and approximates the rate for the United States. A number of countries achieved an “AIDS death to treatment tipping point” ratio of less than 1 whereby more people are put on ART than those estimated to be dying in a given year. Specifically, Angola, Haiti, India, Malawi, Mozambique, Myanmar, Namibia, Tanzania, Uganda, South Africa, Zambia, and Zimbabwe had surpassed the estimated annual number of deaths with reported number of people put on ART, with an “AIDS death to treatment tipping point” ratio of less than 1 (**Table 1**). Similarly, 12 of the 30 high-burden countries achieved the “new HIV infections to treatment tipping point” ratio of less than 1. But it is still very high in some countries (>3.5) including Malaysia, Indonesia, Cameroon, Côte d’Ivoire and South Sudan.

**Table 1:** Estimated deaths per 1000 people living with HIV for top 30 countries with the highest burden of estimated AIDS deaths, 2013.

COUNTRY	AIDS-related deaths* (Uncertainty bound)	No. placed on ART in 2013	AIDS-related deaths to treatment ratio	New HIV infections (Uncertainty bound)	New HIV infections to treatment ratio	People on ART [coverage % (Uncertainty bound)]**	PLHIV (Uncertainty bound)	AIDS-related deaths per 1000 PLHIV (Uncertainty bound)	ART eligibility criteria***
Nigeria	210,000 (190,000–240,000)	148,376	1.41	220,000 (180,000–270,000)	1.48	639,397 [20 (18–21)]	3,200,000 (3,000,000–3,600,000)	65 (63–68)	<350
South Africa	200,000 (170,000–220,000)	472,391	0.41	340,000 (310,000–370,000)	0.72	2,623,271 [42 (40–44)]	6,300,000 (6,000,000–6,500,000)	31 (29–33)	<500
India	130,000 (93,000–160,000)	142,188	0.89	130,000 (80,000–250,000)	0.91	747,175 [36 (28–45)]	2,100,000 (1,700,000–2,700,000)	61 (56–62)	<350
Mozambique	82,000 (70,000–98,000)	187,604	0.44	120,000 (92,000–150,000)	0.64	497,455 [32 (28–36)]	1,600,000 (1,400,000–1,800,000)	53 (50–55)	<350
United Republic of Tanzania	78,000 (69,000–90,000)	81,660	0.96	72,000 (59,000–87,000)	0.88	512,555 [37 (34–40)]	1,400,000 (1,300,000–1,500,000)	56 (53–59)	<500
Zimbabwe	64,000 (59,000–68,000)	99,624	0.64	69,000 (61,000–78,000)	0.69	665,299 [48 (46–50)]	1,400,000 (1,300,000–1,400,000)	46 (44–47)	<500
Uganda	63,000 (56,000–71,000)	156,633	0.40	140,000 (120,000–160,000)	0.89	595,175 [38 (36–40)]	1,600,000 (1,500,000–1,700,000)	40 (38–43)	<500
Kenya	58,000 (49,000–72,000)	52,369	1.12	100,000 (79,000–130,000)	1.91	656,369 [41 (38–45)]	1,600,000 (1,500,000–1,700,000)	37 (33–41)	<500
Malawi	48,000 (44,000–52,000)	67,734	0.71	34,000 (28,000–41,000)	0.50	472,865 [46 (44–49)]	1,000,000 (970,000–1,100,000)	47 (45–48)	<500
Ethiopia	45,000 (36,000–55,000)	29,306	1.54	21,000 (15,000–32,000)	0.72	317,443 [40 (36–44)]	790,000 (720,000–890,000)	57 (51–62)	<500
Cameroon	44,000 (40,000–48,000)	8,811	4.95	47,000 (39,000–55,000)	5.33	131,594 [22 (20–23)]	600,000 (560,000–650,000)	72 (70–75)	<500
Democratic Republic of the Congo	30,000 (24,000–38,000)	15,759	1.91	34,000 (27,000–42,000)	2.16	79,978 [18 (15–21)]	440,000 (370,000–520,000)	68 (64–73)	<500
Indonesia	29,000 (17,000–46,000)	22,489	1.29	80,000 (49,000–170,000)	3.56	54,144 [8 (5–13)]	640,000 (420,000–1,000,000)	45 (41–46)	<350
Côte d'Ivoire	28,000 (25,000–32,000)	2,995	9.33	19,000 (12,000–26,000)	6.34	112,920 [30 (27–34)]	370,000 (330,000–410,000)	75 (74–77)	<350
Zambia	27,000 (23,000–32,000)	99,166	0.27	54,000 (46,000–64,000)	0.54	580,091 [52 (49–55)]	1,100,000 (1,100,000–1,200,000)	24 (22–27)	<500
Thailand	18,000 (16,000–21,000)	7,318	2.52	8,200 (4,000–17,000)	1.12	246,049 [57 (52–62)]	440,000 (400,000–470,000)	42 (41–45)	HIV diagnosis
Lesotho	16,000 (15,000–18,000)	9,852	1.64	26,000 (23,000–30,000)	2.64	101,635 [28 (26–29)]	360,000 (350,000–380,000)	44 (42–47)	<500
Brazil	16,000 (12,000–21,000)	NA	NA	44,000 (35,000–58,000)	NA	NA	730,000 (660,000–810,000)	22 (18–26)	HIV diagnosis
Chad	15,000 (12,000–18,000)	5,632	2.60	12,000 (9,000–16,000)	2.13	42,158 [21 (17–24)]	210,000 (170,000–250,000)	71 (68–74)	NA
Ukraine	13,000 (10,000–18,000)	NA	NA	8,600 (5,500–14,000)	NA	NA	210,000 (180,000–250,000)	64 (58–69)	<200 (<350 reported)
South Sudan	13,000 (4,800–29,000)	1,987	6.34	15,000 (4,800–39,000)	7.55	6,899 [5 (2–12)]	150,000 (59,000–350,000)	82 (81–83)	<500
Viet Nam	12,000 (9,800–16,000)	9,976	1.21	14,000 (11,000–21,000)	1.40	82,687 [33 (30–36)]	250,000 (230,000–280,000)	49 (43–56)	<350
Angola	12,000 (6,000–18,000)	15,491	0.74	28,000 (18,000–42,000)	1.81	64,905 [26 (19–36)]	250,000 (180,000–340,000)	46 (35–52)	<350 (<500 reported)
Central African Republic	11,000 (9,500–12,000)	(64)		7,700 (6,200–9,400)		16,568 [14 (12–16)]	120,000 (110,000–130,000)	91 (89–93)	NA (<500 reported)
Myanmar	11,000 (8,600–12,000)	13,934	0.75	6,700 (5,300–8,400)	0.48	67,643 [35 (31–40)]	190,000 (170,000–220,000)	55 (51–57)	<500
Ghana	10,000 (5,000–18,000)	5,892	1.71	7,800 (2,100–17,000)	1.32	75,762 [34 (25–44)]	220,000 (170,000–300,000)	45 (29–59)	<350 (<500 reported)
Togo	6,600 (2,400–15,000)	4,178	1.59	3,900 (730–9,700)	0.93	34,489 [30 (18–51)]	110,000 (67,000–190,000)	59 (35–75)	NA
Namibia	6,600 (4,000–10,000)	10,932	0.60	12,000 (8,600–16,000)	1.10	126,779 [52 (43–61)]	250,000 (210,000–290,000)	27 (19–35)	<500
Haiti	6,400 (5,500–7,700)	11,516	0.56	6,700 (5,400–8,300)	0.58	54,745 [39 (36–43)]	140,000 (130,000–150,000)	46 (43–51)	<350
Malaysia	5,900 (4,100–8,900)	2,285	2.58	8,000 (5,500–12,000)	3.50	17,369 [20 (15–26)]	86,000 (66,000–120,000)	68 (63–77)	<350
North America and Western Europe****									
Canada (2011)	420 (370–530)	NA	NA	NA	NA	NA	71,300 (58,600–84,000)^1^	5.9 (5–6.3)	<350
France	1,500 (1,100–1,800)	NA	NA	6,900 (2,800–11,000)	NA	NA	NA	NA	HIV diagnosis
Germany (2012)	420 (380–470)	NA	NA	NA	NA	NA	78,000 (70,000–88,000)	5.4 (5.3–5.4)	Consider >500
Italy (2012)	NA (1,200–1,700)	NA	NA	NA	NA	NA	NA (110,000–140,000)	NA (11–12)	Consider >500
The Netherlands (2012)	NA (<200-<200)	NA	NA	NA	NA	NA	NA (20,000–34,000)	NA (6–10)	HIV diagnosis
Spain	800 (720–890)	NA	NA	3,300 (2,200–4,300)	NA	101,542 [68(63–78)]^2^	150,000 (130,000–160,000)	5.4 (5.3–5.4)	HIV diagnosis
United Kingdom	580 (460–750)	NA	NA	6,800 (4,800–9,300)	NA	NA	130,000 (100,000–160,000)	4.6 (4.6–4.7)	<350
United States of America (2011)	20,000 (16,000–28,000)	NA	NA	49,000 (17,000–110,000)	NA	NA	1,300,000 (1,000,000–2,000,000)	15 (14–16)	HIV diagnosis

*The mortality estimate methodology is fully described elsewhere and takes into consideration parameters such as ART coverage. For example, HIV associated mortality in Mozambique also reflects injection drug user driven epidemic.

**ART coverage calculated using 2013 reported people on ART/people estimated to be living with HIV in 2013.

*** Published guidelines as of December 2014; WHO 2013 Guidelines recommend <500 CD4 cell count or irrespective of CD4 cell count for TB patients, pregnant women, children less than 5 years of age, HIV positive person in a serodiscordant couple and/or Hepatitis B.^13^ Number refers to CD4 cell count eligibility criteria for ART. *HIV diagnosis* signifies that treatment can be offered without requiring CD4 cell count and/or other diagnostic criteria for starting treatment (i.e., “test and treat”).

**** Estimates for deaths and people living with HIV for 2012 from UNAIDS Report on the Global AIDS Epidemic, 2013^2^ and for 2011 from UNAIDS Report on the Global AIDS Epidemic, 2012 Other data from UNAIDS 2014 Gap Report^3^ and UNAIDS AIDSinfo data tool.^18^

^1^Public Health Agency of Canada. Summary: Estimates of HIV Prevalence and Incidence in Canada, 2011. Available from: http://www.phac-aspc.gc.ca/aids-sida/publication/survreport/estimat2011-eng.php.

2Ministry of Health, Social Services and Equality, Spain. UNAIDS Country Progress report 2014. Available from: http://www.unaids.org/en/dataanalysis/knowyourresponse/countryprogressreports/2014countries/ESP_narrative_report_2014.pdf.

### Trends in AIDS-related deaths, new HIV infections and ART coverage in Nigeria and South Africa

**Fig 4:**
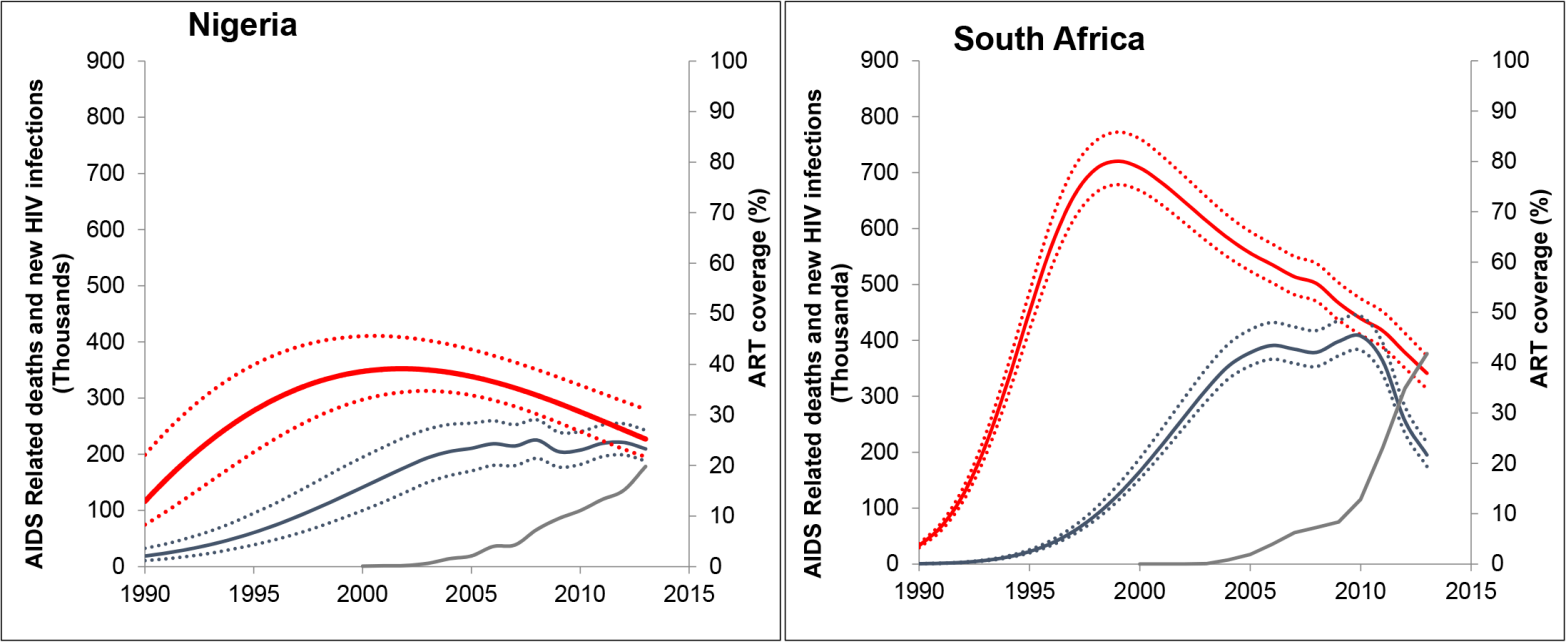
Trends in estimated new infections, AIDS-related deaths and ART coverage in Nigeria and South Africa, 1990–2013. Trends with uncertainty bounds for new infections (red), AIDS-related deaths (blue), and ART coverage percentage (green), 1990 to 2013. ART coverage is calculated as the percentage of people reported on ART among the estimated people living with HIV for the same time period.

Nigeria and South Africa account for 27% of the global HIV burden and have the highest AIDS-related death burden (**Table 1**). **Fig 4**plots the trends in AIDS-related deaths, new HIV infections, and ART coverage in Nigeria and South Africa from 1990–2013. After a peak in new HIV infections in 1999 in South Africa and 2000 in Nigeria, a declining trend has been observed in both countries. In 2000, new HIV infections in South Africa and Nigeria were 710,000 (670,000–760,000) and 360,000 (320,000–420,000), respectively, decreasing to 340,000 (310,000–370,000) and 220,000 (180,000–270,000) in 2013, respectively. While AIDS-related deaths peaked at 410,000 (380,000–440,000) in 2010 in South Africa, they have shown an increasing trend in Nigeria. From 2010–2013, South Africa has experienced a 52% decline in AIDS-related deaths while there was no decline in Nigeria. ART scale-up has progressed more slowly in Nigeria when compared to South Africa, especially between 2010 and 2013. In 2013, 42% (40%-44%) of the people living with HIV were on ART in South Africa, compared to ART coverage of 13% (12%-13%) in 2010. ART coverage in 2013 was 20% (18%-21%) in Nigeria, up from 11% (10%-12%) in 2010. South Africa has a new HIV infection to treatment ratio of 0.72 while Nigeria’s annual ratio is approximately 1.5 new HIV infections for each person placed on treatment.

### ART coverage and AIDS-related deaths projections

**Fig 5:**
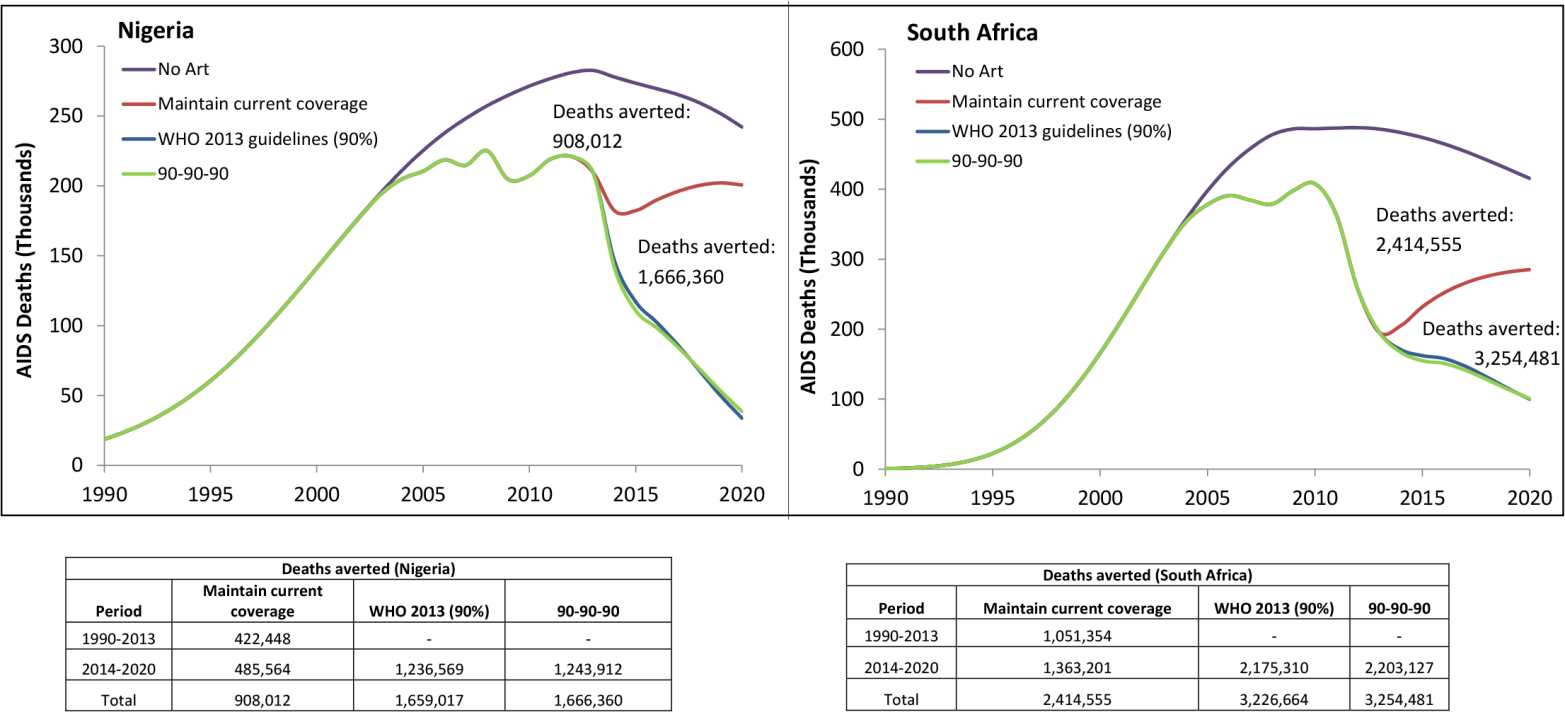
Projected estimated deaths averted by ART coverage scenarios for South Africa and Nigeria, 1990–2020. Projected deaths averted by ART expansion scenarios for South Africa and Nigeria. Reference baseline is projected deaths in the absence of ART (*No ART scenario*) which is compared to 1) maintenance of current ART coverage levels till 2020 (*Current ART Coverage scenario*); 2) expansion of access to 90% ART coverage rates of people eligible under WHO 2013 guidelines by 2020 (*2013 WHO Guidelines scenario*) and 3) UNAIDS “90-90-90” target or 81% of people living with HIV on ART by 2020 (*90-90-90 Scenario)*. The estimated deaths averted represent the total difference from the *No ART scenario*.

Between 1990 and 2013, ART has averted nearly 1 million and 0.4 million AIDS-related deaths in South Africa and Nigeria, respectively. A projection of estimated deaths in the *Current ART Coverage Scenario* suggests that maintaining the current levels of ART coverage through 2020 would avert 1.3 and 0.5 million deaths between 2014 and 2020 for South Africa and Nigeria, respectively, compared to the *No ART Scenario* (**Fig 5**). Compared to the *Current ART Coverage Scenario*, increasing ART coverage to the reach the UN 90-90-90 Target (i.e. 90-90-90 *Scenario*) would avert an estimated 2.2 million and 1.2 million deaths for South Africa and Nigeria, respectively. The projections for 2020 suggest that the 90-90-90 *Scenario* averts a similar number of deaths as the *2013 WHO Guidelines Scenario* (90% ART coverage among those people who are eligible for ART by 2020).

## Discussion

After more than 30 years, almost 77 million people have been infected with HIV and about 39 million have died of AIDS-related causes.[3] The global community has made considerable progress in addressing this unprecedented epidemic. The estimated number of annual AIDS-related deaths has decreased 38% from 2.4 million (2.2 million–2.6 million) in 2005 to 1.5 million (1.4 million–1.7 million) in 2013. This reflects the decline in annual new HIV infections, which peaked at 3.7 million (3.4 million–3.9 million) nearly a decade earlier.[18] The decline also reflects the ART prevention impact on illness, death, transmission and costs; indeed, evidence is accumulating that people who access ART early in the course of HIV infection may live a near-normal lifespan.[4,5,7–9, 20, 21] ART has other benefits including reducing the individual risk of developing TB by 65%, improving employment for individuals and preventing significant societal and health sector costs.[6, 21–24] Globally, access to treatment has expanded from thousands of people in 2000 to 12.9 million reported on ART in 2013.[3] Although these gains are impressive and we are likely to reach the target of 15 million people on ART by end of 2015, our analyses found very high death rates in many settings, low ART coverage, and significantly high rates of AIDS-related deaths. These findings are grounds for serious concern and for re-thinking our current approach to preventing AIDS-related mortality.[14,16]

There are significant variations in the regional burden of AIDS-related deaths, with sub-Saharan Africa and Asia and the Pacific encompassing 90% of the global HIV mortality burden. Treatment access has increased in these regions, and since 2005 they have seen a decline of 39% and 26% decline in deaths, respectively. This positive trend is contrasted by the situation in the Middle East and North Africa, which witnessed a 66% increase in estimated AIDS deaths between 2005 and 2013. Further analyses illustrate the variation and concentration of AIDS-related deaths in limited geographic settings. Eight countries, including only one outside of sub-Saharan Africa (India), account for over 50% of global AIDS-related deaths. Additionally, the top two countries account for 27% of global mortality further highlighting the strategic importance of focusing efforts for maximal impact.

Each of the estimated 35 million people living with HIV will eventually need treatment for their own health, and the time from infection to ART eligibility is a matter of months to a few years.[25] Access to ART has a direct impact on an individual’s risk of death, and the country where one lives has a significant impact on death rates and life expectancy. Our estimates illustrate the unacceptably high annual AIDS-related death rate in many geographic settings and the growing divide between those with and those without earlier access to life-saving ART. In some lower income countries, people living with HIV have 10 to 20 times higher death rates than those living in some higher income countries. Indeed, the stark differences in annual death rates between higher income countries in North America and Western Europe and lower income countries raise important ethical, equity and human rights issues. These markedly different annual risks related to differences in early access to ART can also be found between heavily burdened countries in low- and middle-income categories.

Assuming a life expectancy of 10 years after HIV infection without ART, the estimated annual death rate would be about 100 people per 1000 living with HIV. Our examination of the two countries with the largest number of annual AIDS-related deaths highlights both the marked geographic differences between our response and the grave implications of chronically inadequate ART coverage. As part of its comprehensive response, South Africa has invested considerable resources to improve access to HIV testing and treatment for people living with HIV and has reached nearly 42% (40%-44%) coverage in 2013. Additionally South Africa recently endorsed the UNAIDS 90-90-90 Target and has deployed viral load testing to measure viral suppression.[26] In this setting of rapidly expanded access to HIV testing, ART and access to viral load testing, the estimated number of AIDS-related deaths and new HIV infections are sharply declining. However, this is contrasted by the estimations for Nigeria, which suggest that while ART coverage is increasing slowly and remains below 20% (18%-21%) and new infections are declining, the number of AIDS-related deaths is increasing. We project that South Africa and Nigeria could avert millions of deaths through the expansion of ART access, however, the number of deaths averted will directly depend on whether people living with HIV have earlier access to ART. These two examples illustrate the considerable potential impact of ART but also highlight that in many settings we have not realized the full benefits of ART access expansion.

Nigeria and South Africa are not alone in their efforts to expand access to ART to avert illness, death and transmission. Of the other 30 high-burden countries, 12 have achieved the “AIDS death to treatment tipping point” whereby the number of people being placed on ART in 2013 outstripped the number of annual AIDS-related deaths. For example, through the rapid expansion of access to HIV testing and ART, Botswana has reduced its estimated AIDS-related death rate nearly 75% to levels commensurate with the United States. Botswana is heavily impacted by HIV and has one of the highest burdens of HIV globally. In 2003, HIV prevalence among women attending antenatal clinics was 40%[27] and in 2004 a national household survey found HIV prevalence to be 28% in adult women and 20% in adult men.[28] In 2005, there were an estimated 280,000 persons 15 years and older living with HIV and by 2013, this estimate increased to 310,000.[3,18] In 2002, the Government of Botswana launched the national ART program with the goal of making ART freely available to all eligible persons living with HIV.[29] At the end of 2003, there were 11,000 adults 15 years and older on ART; by 2013 this had increased to 210,000 with estimated ART coverage level among adults of 69%.[3,18] Between 2005 and 2013, the estimated annual new HIV infections decreased by 39% from 15,000 to 9,100 while the estimated annual AIDS-related deaths decreased by 59% from 14,000 to 9,100. [3,18]

To turn the tide on the HIV epidemic and reduce the total number of people living with HIV, new HIV infections need to be prevented through improved access to a continuum of HIV services, including ART.[11] For successful HIV elimination, new infections reflecting the basic reproductive number (R_O_), or transmissions from a person living with HIV within their lifetime, will need to be below 1 and less than the annual number of people accessing treatment.[11,19,30]. In sub-Sahara African HIV epidemics the median value of R_O_ is 4.6 and in all but 5 countries R_O_ is less than 6.3.[31] If the recently proposed UN 90-90-90 Target is achieved, this should reach elimination (R_O_ <1) in 70% of all countries in sub-Saharan Africa and will reduce R_O_ to less than 2 in the remaining 12 countries, making elimination easy to achieve by other high-impact prevention methods.[31] Our results highlight that an accelerated response including ART to decrease transmission is needed to “turn off the tap” and move towards ending the epidemic.

Although ART initiation guidelines are only one element of national ART programs, it is likely that mortality rates among people living with HIV will be higher in countries that recommend that people defer treatment until they are severely immune-compromised below at a CD4 count of <350 cells/mm^3^ and/or they present in clinic with an AIDS-defining illness(es).[4,20,32] Reported and published national ART initiation guidelines for the 30 high-burden countries illustrate that although Brazil and Thailand recommend ART initiation irrespective of CD4 count, only 14 countries representing 49% of the global HIV burden have adopted the WHO 2013 ART initiation criteria.[33,34] Recommending earlier ART eligibility is only part of the solution for people who need ART and successful programmes have also included accelerated efforts to ensure early access to HIV diagnoses, delivery of results to people living with HIV and linkage to care services.

Our study has a number of limitations. Mortality may have multiple causes and the attribution to HIV or other etiologies is difficult, particularly in low- and middle-income countries. Few countries monitor and report annual AIDS-related deaths and we rely on the modelled AIDS-related deaths estimation instead of the actual number of reported deaths among people living with HIV. Recent studies reflect an ongoing discussion regarding the best estimations for mortality-related model parameters [35] and have questioned the assumptions agreed upon by the UNAIDS Reference Group for Estimates, Modelling and Projections.[36] However, our results are directionally consistent with these new studies and show similar trends in AIDS deaths.[35,36] The strong relationship between ART coverage and the prevention of AIDS-related mortality is both logical and scientifically sound. Since ART coverage is a component in the UNAIDS mortality estimations, it was not meaningful to measure the direct association in our study. Additionally, the number of people on ART is based on values collected by countries that have their own limitations such as exclusion of persons receiving ART in the private sector, [37] ability to disaggregate by age, potential double counting when individuals move between clinics and others. Our article presents in-depth analyses of a few high-burden countries; however, it is beyond the scope of this study to expand the analyses to other settings. While regional- and country-level estimates are important for benchmarking progress and strategic planning, there would clearly be additional value for mapping sub-national responses and mortality. Our work is incomplete in that it does not report on ART and mortality burdens among marginalized and vulnerable populations such as the urban and rural poor, [38] sex workers, [39] men who have sex with men, [40] drug users, [41] pregnant women and children. [42] Improving data collection regarding ART coverage for these important key populations would improve the feasibility of estimating progress towards reducing the impact of HIV on incidence, prevalence and mortality.

The global response to the HIV epidemic has been remarkable and has averted millions of premature deaths. [3,14,16] However, despite these impressive gains, our study illustrates the continuing significant negative impact of HIV and the missed public health opportunity to scale up HIV testing, ART access, and facilitate viral suppression for those people on ART to prevent millions of additional deaths, particularly in low- and middle-income countries. Although we did not examine the impact on other morbidity, HIV transmission or cost savings, it is nevertheless clear that expanding access to earlier ART beyond the currently reported 14 (40%) million people [43] will have significant individual and public health impact. Additionally, the geographic disparity in mortality trends and rates illustrates the need for further focus and rapid action to reduce AIDS-related deaths in those countries where the problem is most severe. In many settings, realizing this potential will require a transformation of traditional service delivery models to include more community-based and-led services and a focus on greater equity, including ensuring that key populations have access to HIV services and earlier ART. Innovative strategies to focus efforts on ending AIDS where HIV epidemics are most concentrated will be needed to expand access beyond current approaches. Recognizing the significant potential to end AIDS in the highest HIV burden cities in the world, the International Association of Providers of AIDS Care (IAPAC), in partnership with UNAIDS, UN-Habitat and Mayors from various cities around the world, has recently launched the Fast-Track Cities Initiative which will focus on rapidly achieving the UN 90-90-90 Target as a means to end the AIDS pandemic in these urban settings.[15,44] Despite progress in expanding access to treatment, high-burden cities, regions, and countries, with ongoing support from partners including the President's Emergency Plan for AIDS Relief (PEPFAR) and the Global Fund to Fight AIDS, Tuberculosis, and Malaria, must continue to accelerate access to testing and treatment, within the context of robust HIV responses, to avert millions more premature AIDS deaths and sustainably reduce new HIV infections.
